# Tetra­aqua­bis(isonicotinamide-κ*N* ^1^)nickel(II) bis­(4-formyl­benzoate) dihydrate

**DOI:** 10.1107/S1600536809032279

**Published:** 2009-08-19

**Authors:** Tuncer Hökelek, Filiz Yılmaz, Barış Tercan, Ferdi Gürgen, Hacali Necefoğlu

**Affiliations:** a Department of Physics, Hacettepe University, 06800 Beytepe, Ankara, Turkey; b Department of Chemistry, Faculty of Science, Anadolu University, 26470 Yenibağlar, Eskişehir, Turkey; c Department of Physics, Karabük University, 78050 Karabük, Turkey; d Department of Chemistry, Kafkas University, 63100 Kars, Turkey

## Abstract

The asymmetric unit of the title complex, [Ni(C_6_H_6_N_2_O)_2_(H_2_O)_4_](C_8_H_5_O_3_)_2_·2H_2_O, contains one-half of the complex cation with the Ni^II^ atom located on an inversion center, a 4-formyl­benzoate (FB) counter-anion and an uncoordinated water mol­ecule. The four O atoms in the equatorial plane around the Ni atom form a slightly distorted square-planar arrangement and the slightly distorted octa­hedral coordination is completed by the two N atoms of the isonicotinamide (INA) ligands at a sligthly longer distance in the axial positions. The dihedral angle between the carboxyl­ate group and the attached benzene ring is 8.14 (11)°, while the pyridine and benzene rings are oriented at a dihedral angle of 3.46 (6)°. In the crystal structure, O—H⋯O, N—H⋯O and C—H⋯O hydrogen bonds link the mol­ecules into a three-dimensional network. π–π Contacts between the benzene and pyridine rings [centroid–centroid distance = 3.751 (1) Å] may further stabilize the crystal structure.

## Related literature

For general background, see: Bigoli *et al.* (1972 ▶); Krishnamachari (1974 ▶). For related structures, see: Hökelek *et al.* (2009 ▶); Sertçelik *et al.* (2009 ▶).
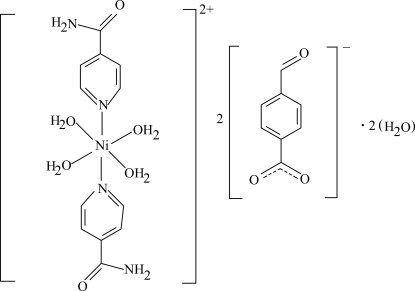


## Experimental

### 

#### Crystal data

[Ni(C_6_H_6_N_2_O)_2_(H_2_O)_4_](C_8_H_5_O_3_)_2_·2H_2_O
*M* *_r_* = 709.28
Triclinic, 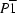

*a* = 6.4338 (1) Å
*b* = 6.9059 (2) Å
*c* = 18.1649 (3) Å
α = 81.658 (2)°
β = 85.160 (3)°
γ = 71.816 (1)°
*V* = 758.01 (3) Å^3^
*Z* = 1
Mo *K*α radiation
μ = 0.72 mm^−1^
*T* = 100 K
0.35 × 0.15 × 0.10 mm

#### Data collection

Bruker Kappa APEXII CCD area-detector diffractometer
Absorption correction: multi-scan (*SADABS*; Bruker, 2005 ▶) *T*_min_ = 0.876, *T*_max_ = 0.928
14080 measured reflections
3837 independent reflections
3259 reflections with *I* > 2σ(*I*)
*R*_int_ = 0.067

#### Refinement

*R*[*F* ^2^ > 2σ(*F* ^2^)] = 0.033
*wR*(*F* ^2^) = 0.080
*S* = 1.01
3837 reflections
250 parameters
H atoms treated by a mixture of independent and constrained refinement
Δρ_max_ = 0.50 e Å^−3^
Δρ_min_ = −0.47 e Å^−3^

### 

Data collection: *APEX2* (Bruker, 2007 ▶); cell refinement: *SAINT* (Bruker, 2007 ▶); data reduction: *SAINT*; program(s) used to solve structure: *SHELXS97* (Sheldrick, 2008 ▶); program(s) used to refine structure: *SHELXL97* (Sheldrick, 2008 ▶); molecular graphics: *ORTEP-3 for Windows* (Farrugia, 1997 ▶); software used to prepare material for publication: *WinGX* (Farrugia, 1999 ▶) and *PLATON* (Spek, 2009 ▶).

## Supplementary Material

Crystal structure: contains datablocks I, global. DOI: 10.1107/S1600536809032279/xu2589sup1.cif

Structure factors: contains datablocks I. DOI: 10.1107/S1600536809032279/xu2589Isup2.hkl

Additional supplementary materials:  crystallographic information; 3D view; checkCIF report

## Figures and Tables

**Table 1:** Selected bond lengths (Å)

Ni1—O5	2.0444 (11)
Ni1—O6	2.0857 (11)
Ni1—N1	2.0978 (12)

**Table 2:** Hydrogen-bond geometry (Å, °)

*D*—H⋯*A*	*D*—H	H⋯*A*	*D*⋯*A*	*D*—H⋯*A*
N2—H21⋯O7^i^	0.84 (2)	2.03 (2)	2.8518 (19)	163.7 (17)
N2—H22⋯O3^ii^	0.879 (19)	2.109 (18)	2.9662 (17)	165.1 (18)
O5—H51⋯O1^iii^	0.78 (3)	1.87 (2)	2.6485 (16)	177 (3)
O5—H52⋯O2	0.897 (19)	1.847 (19)	2.7349 (16)	169.6 (18)
O6—H61⋯O1^iv^	0.87 (3)	1.91 (3)	2.7792 (17)	175 (2)
O6—H62⋯O2^v^	0.84 (3)	1.94 (3)	2.7710 (17)	171 (3)
O7—H71⋯O3^iii^	0.81 (3)	2.11 (3)	2.9260 (19)	176 (2)
O7—H72⋯O4	0.84 (2)	1.90 (2)	2.7333 (18)	174 (2)
C9—H9⋯O7^i^	0.93	2.59	3.480 (2)	160
C12—H12⋯O5^vi^	0.93	2.44	3.193 (2)	138

Symmetry codes: (i) 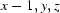; (ii) 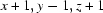; (iii) 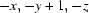; (iv) 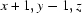; (v) 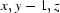; (vi) 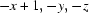.

**Fig. 1.**
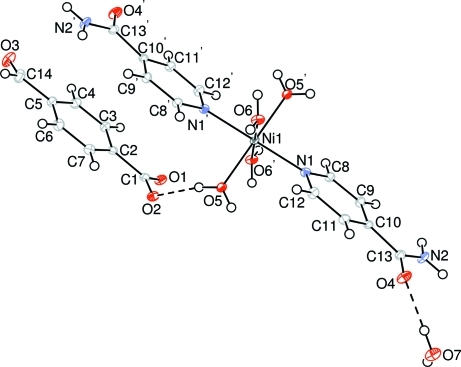
The molecular structure of the title molecule with the atom-numbering scheme. Displacement ellipsoids are drawn at the 50% probability level. Primed atoms are generated by the symmetry operator:(') -x, -y, -z. Dashed lines indicate the hydrogen bonding.

## supplementary crystallographic information

### Comment

As a part of our ongoing investigation on transition metal complexes of nicotinamide (NA), one form of niacin (Krishnamachari, 1974), and/or the nicotinic acid derivative *N*,*N*-diethylnicotinamide (DENA), an important respiratory stimulant (Bigoli *et al.*, 1972), the title compound was synthesized and its crystal structure is reported herein.

The title compound is a monomeric complex, with Ni^II^ ion on a centre of symmetry. The structures of some DENA and/or NA complexes of Ni^II^ ion, [Ni(C_8_H_5_O_3_)_2_(C_10_H_14_N_2_O)_2_(H_2_O)_2_] (Sertçelik *et al.*, 2009) and [Ni(C_7_H_4_BrO_2_)_2_(C_6_H_6_N_2_O)_2_(H_2_O)_2_] (Hökelek *et al.*, 2009) have also been determined.

In the title compound, INA ligands are monodentate. The four O atoms (O5, O6, and the symmetry-related atoms, O5', O6') in the equatorial plane around the Ni atom form a slightly distorted square-planar arrangement, while the slightly distorted octahedral coordination is completed by the two pyridine N atoms (N1, N1') of the INA ligands at 2.0978 (12) Å from the Ni atom in the axial positions (Table 1, Fig. 1). The average Ni—O bond length is 2.0651 (11) Å. The O—H···O hydrogen bonds (Table 2) link the coordinated and uncoordinated water molecules to the FB anion and INA ligand, respectively. The dihedral angle between the carboxyl group (O1/O2/C1) and the benzene ring A (C2—C7) is 8.14 (11)°, while that between rings A and B (N1/C8—C12) is 3.46 (6)°.

In the crystal structure, the O—H···O, N—H···O and C—H···O hydrogen bonds (Table 2) link the molecules into a three-dimensional network, in which they may be effective in the stabilization of the structure. The π–π contact between the benzene and pyridine rings, *Cg*1—*Cg*2, [where *Cg*1 and *Cg*2 are centroids of the rings A (C2—C7) and B (N1/C8—C12), respectively] may further stabilize the structure, with centroid-centroid distance of 3.751 (1) Å.

### Experimental

The title compound was prepared by the reaction of NiSO_4_.6H_2_O (1.31 g, 5 mmol) in H_2_O (25 ml) and INA (1.22 g, 10 mmol) in H_2_O (40 ml) with sodium 4-formylbenzoate (1.72 g, 10 mmol) in H_2_O (50 ml). The mixture was filtered and set aside to crystallize at ambient temperature for several days, giving blue single crystals.

### Refinement

Atoms H51, H52, H61, H62, H71 and H72 (for H_2_O), H21 and H22 (for NH_2_) and H14 (for CH) were located in difference Fourier map and refined isotropically. The remaining H atoms were positioned geometrically with C—H = 0.93 Å, for aromatic H atoms and constrained to ride on their parent atoms, with *U*_iso_(H) = 1.2*U*_eq_(C).

### Crystal data

**Table:**

[Ni(C_6_H_6_N_2_O)_2_(H_2_O)_4_](C_8_H_5_O_3_)_2_·2H_2_O	*Z* = 1
*M**_r_* = 709.28	*F*(000) = 370
Triclinic, *P*1	*D*_x_ = 1.554 Mg m^−^^3^
Hall symbol: -P 1	Mo *K*α radiation, λ = 0.71073 Å
*a* = 6.4338 (1) Å	Cell parameters from 5282 reflections
*b* = 6.9059 (2) Å	θ = 4.1–28.3°
*c* = 18.1649 (3) Å	µ = 0.72 mm^−^^1^
α = 81.658 (2)°	*T* = 100 K
β = 85.160 (3)°	Block, blue
γ = 71.816 (1)°	0.35 × 0.15 × 0.1 mm
*V* = 758.01 (3) Å^3^	

### Data collection

**Table:**

Bruker Kappa APEXII CCD area-detector diffractometer	3837 independent reflections
Radiation source: fine-focus sealed tube	3259 reflections with *I* > 2σ(*I*)
graphite	*R*_int_ = 0.067
φ and ω scans	θ_max_ = 28.5°, θ_min_ = 2.3°
Absorption correction: multi-scan (*SADABS*; Bruker, 2005)	*h* = −8→8
*T*_min_ = 0.876, *T*_max_ = 0.928	*k* = −9→9
14080 measured reflections	*l* = −24→24

### Refinement

**Table:**

Refinement on *F*^2^	Primary atom site location: structure-invariant direct methods
Least-squares matrix: full	Secondary atom site location: difference Fourier map
*R*[*F*^2^ > 2σ(*F*^2^)] = 0.033	Hydrogen site location: inferred from neighbouring sites
*wR*(*F*^2^) = 0.080	H atoms treated by a mixture of independent and constrained refinement
*S* = 1.01	*w* = 1/[σ^2^(*F*_o_^2^) + (0.0396*P*)^2^] where *P* = (*F*_o_^2^ + 2*F*_c_^2^)/3
3837 reflections	(Δ/σ)_max_ < 0.001
250 parameters	Δρ_max_ = 0.50 e Å^−^^3^
0 restraints	Δρ_min_ = −0.47 e Å^−^^3^

### Special details

**Table:**

Geometry. All e.s.d.'s (except the e.s.d. in the dihedral angle between two l.s. planes) are estimated using the full covariance matrix. The cell e.s.d.'s are taken into account individually in the estimation of e.s.d.'s in distances, angles and torsion angles; correlations between e.s.d.'s in cell parameters are only used when they are defined by crystal symmetry. An approximate (isotropic) treatment of cell e.s.d.'s is used for estimating e.s.d.'s involving l.s. planes.
Refinement. Refinement of *F*^2^ against ALL reflections. The weighted *R*-factor *wR* and goodness of fit *S* are based on *F*^2^, conventional *R*-factors *R* are based on *F*, with *F* set to zero for negative *F*^2^. The threshold expression of *F*^2^ > σ(*F*^2^) is used only for calculating *R*-factors(gt) *etc*. and is not relevant to the choice of reflections for refinement. *R*-factors based on *F*^2^ are statistically about twice as large as those based on *F*, and *R*- factors based on ALL data will be even larger.

### Fractional atomic coordinates and isotropic or equivalent isotropic displacement parameters (Å^2^)

**Table:**

	*x*	*y*	*z*	*U*_iso_*/*U*_eq_	
Ni1	0.0000	0.0000	0.0000	0.00952 (9)	
O1	−0.32770 (17)	0.69399 (18)	−0.08791 (6)	0.0156 (2)	
O2	0.02696 (17)	0.55211 (17)	−0.11784 (6)	0.0147 (2)	
O3	−0.5543 (2)	0.7444 (2)	−0.46465 (6)	0.0248 (3)	
O4	0.62907 (18)	−0.23630 (19)	0.31701 (6)	0.0187 (3)	
O5	0.18852 (18)	0.19178 (18)	−0.02699 (6)	0.0121 (2)	
H51	0.228 (4)	0.229 (4)	0.0063 (13)	0.050 (8)*	
H52	0.124 (3)	0.303 (3)	−0.0585 (10)	0.027 (5)*	
O6	0.23596 (19)	−0.24606 (18)	−0.04216 (6)	0.0143 (2)	
H61	0.370 (4)	−0.265 (4)	−0.0591 (12)	0.048 (7)*	
H62	0.181 (4)	−0.320 (4)	−0.0624 (12)	0.040 (7)*	
O7	0.8514 (2)	−0.1254 (2)	0.41718 (7)	0.0214 (3)	
H71	0.771 (4)	−0.021 (4)	0.4322 (13)	0.055 (9)*	
H72	0.775 (3)	−0.155 (3)	0.3879 (12)	0.037 (6)*	
N1	0.1326 (2)	−0.0939 (2)	0.10564 (7)	0.0112 (3)	
N2	0.3028 (2)	−0.1740 (2)	0.37981 (7)	0.0153 (3)	
H21	0.166 (3)	−0.153 (3)	0.3816 (10)	0.024 (5)*	
H22	0.369 (3)	−0.199 (3)	0.4221 (10)	0.021 (5)*	
C1	−0.1722 (2)	0.6250 (2)	−0.13329 (8)	0.0129 (3)	
C2	−0.2270 (2)	0.6334 (2)	−0.21321 (8)	0.0120 (3)	
C3	−0.4455 (2)	0.6912 (2)	−0.23337 (8)	0.0130 (3)	
H3	−0.5580	0.7218	−0.1972	0.016*	
C4	−0.4939 (3)	0.7027 (3)	−0.30658 (8)	0.0149 (3)	
H4	−0.6391	0.7411	−0.3198	0.018*	
C5	−0.3256 (3)	0.6570 (3)	−0.36097 (8)	0.0142 (3)	
C6	−0.1081 (3)	0.5964 (3)	−0.34101 (8)	0.0159 (3)	
H6	0.0043	0.5631	−0.3771	0.019*	
C7	−0.0597 (3)	0.5859 (3)	−0.26751 (8)	0.0144 (3)	
H7	0.0855	0.5468	−0.2543	0.017*	
C8	0.0066 (2)	−0.0685 (2)	0.16870 (8)	0.0119 (3)	
H8	−0.1447	−0.0268	0.1651	0.014*	
C9	0.0914 (2)	−0.1014 (2)	0.23847 (8)	0.0126 (3)	
H9	−0.0017	−0.0812	0.2806	0.015*	
C10	0.3172 (2)	−0.1649 (2)	0.24511 (8)	0.0109 (3)	
C11	0.4476 (2)	−0.1990 (3)	0.18050 (8)	0.0133 (3)	
H11	0.5993	−0.2465	0.1828	0.016*	
C12	0.3506 (2)	−0.1620 (3)	0.11290 (8)	0.0139 (3)	
H12	0.4406	−0.1854	0.0702	0.017*	
C13	0.4277 (3)	−0.1948 (2)	0.31741 (8)	0.0130 (3)	
C14	−0.3734 (3)	0.6682 (3)	−0.43942 (9)	0.0197 (4)	
H14	−0.245 (3)	0.614 (3)	−0.4731 (10)	0.021 (5)*	

### Atomic displacement parameters (Å^2^)

**Table:**

	*U*^11^	*U*^22^	*U*^33^	*U*^12^	*U*^13^	*U*^23^
Ni1	0.00852 (14)	0.01296 (17)	0.00766 (14)	−0.00363 (11)	−0.00026 (10)	−0.00235 (11)
O1	0.0131 (5)	0.0221 (7)	0.0133 (5)	−0.0060 (5)	0.0003 (4)	−0.0068 (5)
O2	0.0121 (5)	0.0168 (6)	0.0160 (5)	−0.0042 (5)	−0.0035 (4)	−0.0032 (5)
O3	0.0253 (7)	0.0314 (8)	0.0150 (6)	−0.0030 (6)	−0.0067 (5)	−0.0039 (5)
O4	0.0123 (6)	0.0267 (7)	0.0168 (5)	−0.0033 (5)	−0.0039 (4)	−0.0063 (5)
O5	0.0122 (5)	0.0151 (6)	0.0101 (5)	−0.0055 (5)	−0.0011 (4)	−0.0017 (5)
O6	0.0111 (6)	0.0176 (6)	0.0159 (5)	−0.0048 (5)	0.0010 (4)	−0.0072 (5)
O7	0.0168 (6)	0.0307 (8)	0.0180 (6)	−0.0065 (6)	−0.0018 (5)	−0.0082 (6)
N1	0.0110 (6)	0.0121 (7)	0.0110 (6)	−0.0038 (5)	0.0009 (5)	−0.0034 (5)
N2	0.0139 (7)	0.0222 (8)	0.0098 (6)	−0.0043 (6)	−0.0040 (5)	−0.0028 (6)
C1	0.0153 (8)	0.0106 (8)	0.0149 (7)	−0.0060 (7)	−0.0018 (6)	−0.0024 (6)
C2	0.0139 (7)	0.0094 (8)	0.0138 (7)	−0.0047 (6)	−0.0022 (6)	−0.0022 (6)
C3	0.0123 (7)	0.0128 (8)	0.0133 (7)	−0.0025 (6)	0.0002 (6)	−0.0028 (6)
C4	0.0134 (7)	0.0157 (9)	0.0151 (7)	−0.0028 (7)	−0.0028 (6)	−0.0023 (7)
C5	0.0180 (8)	0.0136 (8)	0.0118 (7)	−0.0056 (7)	−0.0020 (6)	−0.0017 (6)
C6	0.0167 (8)	0.0157 (9)	0.0143 (7)	−0.0035 (7)	0.0026 (6)	−0.0035 (7)
C7	0.0127 (7)	0.0126 (8)	0.0174 (7)	−0.0029 (6)	−0.0017 (6)	−0.0019 (7)
C8	0.0097 (7)	0.0135 (8)	0.0126 (7)	−0.0034 (6)	−0.0007 (5)	−0.0019 (6)
C9	0.0127 (7)	0.0142 (8)	0.0105 (7)	−0.0038 (6)	0.0009 (6)	−0.0022 (6)
C10	0.0129 (7)	0.0085 (8)	0.0117 (7)	−0.0033 (6)	−0.0019 (6)	−0.0021 (6)
C11	0.0103 (7)	0.0149 (8)	0.0146 (7)	−0.0037 (6)	−0.0008 (6)	−0.0021 (6)
C12	0.0113 (7)	0.0172 (9)	0.0125 (7)	−0.0033 (7)	0.0018 (6)	−0.0033 (6)
C13	0.0148 (8)	0.0105 (8)	0.0137 (7)	−0.0026 (6)	−0.0030 (6)	−0.0027 (6)
C14	0.0242 (9)	0.0203 (10)	0.0133 (7)	−0.0047 (8)	0.0008 (7)	−0.0033 (7)

### Geometric parameters (Å, °)

**Table:**

Ni1—O5	2.0444 (11)	C2—C7	1.391 (2)
Ni1—O5^i^	2.0444 (11)	C3—C2	1.401 (2)
Ni1—O6	2.0857 (11)	C3—C4	1.377 (2)
Ni1—O6^i^	2.0857 (11)	C3—H3	0.9300
Ni1—N1	2.0978 (12)	C4—H4	0.9300
Ni1—N1^i^	2.0978 (12)	C5—C4	1.395 (2)
O1—C1	1.2587 (18)	C5—C6	1.393 (2)
O2—C1	1.2616 (18)	C5—C14	1.469 (2)
O3—C14	1.216 (2)	C6—C7	1.383 (2)
O4—C13	1.2359 (18)	C6—H6	0.9300
O5—H51	0.78 (2)	C7—H7	0.9300
O5—H52	0.90 (2)	C8—H8	0.9300
O6—H61	0.86 (2)	C9—C8	1.382 (2)
O6—H62	0.84 (2)	C9—C10	1.391 (2)
O7—H71	0.82 (3)	C9—H9	0.9300
O7—H72	0.84 (2)	C10—C11	1.388 (2)
N1—C8	1.3465 (18)	C10—C13	1.503 (2)
N1—C12	1.3444 (19)	C11—C12	1.379 (2)
N2—C13	1.3316 (19)	C11—H11	0.9300
N2—H21	0.84 (2)	C12—H12	0.9300
N2—H22	0.879 (19)	C14—H14	0.991 (18)
C2—C1	1.511 (2)		
			
O5^i^—Ni1—O5	180.00 (6)	C4—C3—C2	120.18 (14)
O5—Ni1—O6	92.91 (5)	C4—C3—H3	119.9
O5^i^—Ni1—O6	87.09 (5)	C3—C4—C5	120.09 (14)
O5—Ni1—O6^i^	87.09 (5)	C3—C4—H4	120.0
O5^i^—Ni1—O6^i^	92.91 (5)	C5—C4—H4	120.0
O5—Ni1—N1	91.09 (5)	C4—C5—C6	119.94 (14)
O5^i^—Ni1—N1	88.91 (5)	C4—C5—C14	121.06 (14)
O5—Ni1—N1^i^	88.91 (5)	C6—C5—C14	119.00 (14)
O5^i^—Ni1—N1^i^	91.09 (5)	C5—C6—H6	120.1
O6—Ni1—O6^i^	180.00 (8)	C7—C6—C5	119.89 (14)
O6—Ni1—N1	90.55 (5)	C7—C6—H6	120.1
O6^i^—Ni1—N1	89.45 (5)	C2—C7—H7	119.8
O6—Ni1—N1^i^	89.45 (5)	C6—C7—C2	120.35 (14)
O6^i^—Ni1—N1^i^	90.55 (5)	C6—C7—H7	119.8
N1—Ni1—N1^i^	180.00 (3)	N1—C8—C9	123.15 (14)
Ni1—O5—H51	116.0 (17)	N1—C8—H8	118.4
Ni1—O5—H52	112.8 (12)	C9—C8—H8	118.4
H52—O5—H51	108 (2)	C8—C9—C10	119.38 (13)
Ni1—O6—H61	133.3 (16)	C8—C9—H9	120.3
Ni1—O6—H62	112.7 (15)	C10—C9—H9	120.3
H61—O6—H62	109 (2)	C9—C10—C13	124.09 (13)
H71—O7—H72	104 (2)	C11—C10—C9	117.58 (13)
C8—N1—Ni1	121.97 (10)	C11—C10—C13	118.32 (13)
C12—N1—Ni1	120.69 (9)	C10—C11—H11	120.2
C12—N1—C8	116.97 (13)	C12—C11—C10	119.56 (14)
C13—N2—H21	124.0 (12)	C12—C11—H11	120.2
C13—N2—H22	117.6 (12)	N1—C12—C11	123.27 (13)
H21—N2—H22	118.0 (17)	N1—C12—H12	118.4
O1—C1—O2	125.32 (14)	C11—C12—H12	118.4
O1—C1—C2	117.63 (14)	O4—C13—N2	122.48 (14)
O2—C1—C2	117.04 (13)	O4—C13—C10	119.41 (13)
C3—C2—C1	120.57 (13)	N2—C13—C10	118.11 (13)
C7—C2—C1	119.88 (14)	O3—C14—C5	124.44 (15)
C7—C2—C3	119.54 (14)	O3—C14—H14	119.8 (10)
C2—C3—H3	119.9	C5—C14—H14	115.7 (10)
			
O5—Ni1—N1—C8	120.80 (12)	C4—C3—C2—C7	−0.6 (2)
O5^i^—Ni1—N1—C8	−59.20 (12)	C2—C3—C4—C5	0.0 (2)
O5—Ni1—N1—C12	−51.98 (12)	C6—C5—C4—C3	1.0 (2)
O5^i^—Ni1—N1—C12	128.02 (12)	C14—C5—C4—C3	179.97 (15)
O6—Ni1—N1—C8	−146.28 (12)	C4—C5—C6—C7	−1.3 (2)
O6^i^—Ni1—N1—C8	33.72 (12)	C14—C5—C6—C7	179.69 (15)
O6—Ni1—N1—C12	40.95 (12)	C4—C5—C14—O3	11.3 (3)
O6^i^—Ni1—N1—C12	−139.05 (12)	C6—C5—C14—O3	−169.77 (17)
Ni1—N1—C8—C9	−170.46 (12)	C5—C6—C7—C2	0.7 (2)
C12—N1—C8—C9	2.6 (2)	C10—C9—C8—N1	−0.4 (2)
Ni1—N1—C12—C11	170.82 (12)	C8—C9—C10—C11	−2.1 (2)
C8—N1—C12—C11	−2.3 (2)	C8—C9—C10—C13	176.67 (14)
C3—C2—C1—O1	−8.1 (2)	C9—C10—C11—C12	2.4 (2)
C3—C2—C1—O2	173.07 (14)	C13—C10—C11—C12	−176.49 (14)
C7—C2—C1—O1	171.16 (14)	C9—C10—C13—O4	−174.45 (15)
C7—C2—C1—O2	−7.7 (2)	C9—C10—C13—N2	5.2 (2)
C1—C2—C7—C6	−178.93 (14)	C11—C10—C13—O4	4.3 (2)
C3—C2—C7—C6	0.3 (2)	C11—C10—C13—N2	−176.04 (15)
C4—C3—C2—C1	178.61 (14)	C10—C11—C12—N1	−0.2 (2)

Symmetry codes: (i) −*x*, −*y*, −*z*.

### Hydrogen-bond geometry (Å, °)

**Table:**

*D*—H···*A*	*D*—H	H···*A*	*D*···*A*	*D*—H···*A*
N2—H21···O7^ii^	0.84 (2)	2.03 (2)	2.8518 (19)	163.7 (17)
N2—H22···O3^iii^	0.879 (19)	2.109 (18)	2.9662 (17)	165.1 (18)
O5—H51···O1^iv^	0.78 (3)	1.87 (2)	2.6485 (16)	177 (3)
O5—H52···O2	0.897 (19)	1.847 (19)	2.7349 (16)	169.6 (18)
O6—H61···O1^v^	0.87 (3)	1.91 (3)	2.7792 (17)	175 (2)
O6—H62···O2^vi^	0.84 (3)	1.94 (3)	2.7710 (17)	171 (3)
O7—H71···O3^iv^	0.81 (3)	2.11 (3)	2.9260 (19)	176 (2)
O7—H72···O4	0.84 (2)	1.90 (2)	2.7333 (18)	174 (2)
C9—H9···O7^ii^	0.93	2.59	3.480 (2)	160
C12—H12···O5^vii^	0.93	2.44	3.193 (2)	138

Symmetry codes: (ii) *x*−1, *y*, *z*; (iii) *x*+1, *y*−1, *z*+1; (iv) −*x*, −*y*+1, −*z*; (v) *x*+1, *y*−1, *z*; (vi) *x*, *y*−1, *z*; (vii) −*x*+1, −*y*, −*z*.
